# Knowledge of, attitudes toward, and preventive practices relating to cholera and oral cholera vaccine among urban high-risk groups: findings of a cross-sectional study in Dhaka, Bangladesh

**DOI:** 10.1186/1471-2458-13-242

**Published:** 2013-03-19

**Authors:** Tasnuva Wahed, Sheikh Shah Tanvir Kaukab, Nirod Chandra Saha, Iqbal Ansary Khan, Farhana Khanam, Fahima Chowdhury, Amit Saha, Ashraful Islam Khan, Ashraf Uddin Siddik, Alejandro Cravioto, Firdausi Qadri, Jasim Uddin

**Affiliations:** 1 Centre for Vaccine Sciences, icddr,b, Dhaka, Bangladesh; 2 International Vaccine Institute, Seoul, South Korea; 3 Centre for Equity and Health Systems, icddr,b, Dhaka, Bangladesh

**Keywords:** Cholera, Oral cholera vaccine, Knowledge, Perceptions, Healthcare-seeking, Bangladesh

## Abstract

**Background:**

In endemic countries such as Bangladesh, consequences of cholera place an enormous financial and social burden on patients and their families. Cholera vaccines not only provide health benefits to susceptible populations but also have effects on the earning capabilities and financial stability of the family. Community-based research and evaluations are necessary to understand perceptions about and practices of the community relating to cholera and oral cholera vaccines. This may help identify the ways in which such vaccines may be successfully introduced, and other preventive measures can be implemented. The present study assessed the knowledge of, attitudes toward, and preventive practices relating to cholera and oral cholera vaccine among an urban population residing in a high cholera-prone setting in Dhaka, Bangladesh.

**Methods:**

This cross-sectional study was conducted in an area of high cholera prevalence in 15 randomly-selected clusters in Mirpur, Dhaka city. A study team collected data through a survey and in-depth interviews during December 2010–February 2011.

**Results:**

Of 2,830 families included in the final analysis, 23% could recognize cholera as acute watery diarrhea and 16% had ever heard of oral cholera vaccine. About 54% of the respondents had poor knowledge about cholera-related issues while 97% had a positive attitude toward cholera and oral cholera vaccine. One-third showed poor practice relating to the prevention of cholera.

The findings showed a significant (p < 0.05) association between the respondents’ knowledge and sex, education, occupation, monthly overall household expenditure, attitudes and practice. In the adjusted model, male sex, having a lower monthly overall household expenditure, and having a less positive attitude toward cholera were the significant predictors to having poor knowledge.

**Conclusions:**

The findings suggest the strengthening of health education activities to improve knowledge on cholera, its prevention and treatment and information on cholera vaccination among high-risk populations. The data also underscore the potential of mass cholera vaccination to prevent and control cholera.

## Background

Cholera is a waterborne, life-threatening form of dehydrating diarrheal disease caused by the toxigenic serogroup strains of *Vibrio cholerae*. It remains a public-health threat, as evidenced by its substantial contribution to morbidity and mortality in low-income countries. Globally, about 317,534 cholera cases were reported during 2008–2010 [1,2], with a 52% increase in deaths, half of which occurred in children aged less than five years [3]. However, in real terms, the numbers are likely much higher due to underreporting, differing definitions of acute watery diarrhea from country to country, inconsistencies in case definitions, and poor surveillance systems [4]. Although published government data are not available in Bangladesh, data presented by government personnel showed that there are approximately 450,000 cholera cases annually in Bangladesh [5]. Results of a study by Chowdhury *et al.* showed that 23% of patients who presented to the icddr,b (International Centre for Diarrhoeal Disease Research, Bangladesh), known locally as the “cholera hospital”, were from urban lower socioeconomic groups [6].

A review of literature showed that water source contamination (29%), rainfall and flooding (25%), and refugee settings (13%) are the most common risk factors for cholera worldwide [7]. Another study identified that sharing of a latrine with three or more households is another important risk factor for cholera [8]. A recent study in Cameroon illustrated that low educational activities dedicated to health, hygiene and sanitation practices and an abundance of adulterated food, impact cholera infection and transmission and suggests that behavioral change within the whole community is needed [9]. Results of studies in Bangladesh showed that proximity to surface water, high population density, poor educational level, water and air temperature, and total rainfall were the predictors of cholera cases [10,11].

Treatment of cholera patients imposes a significant financial and social burden on healthcare systems in cholera-endemic countries. Costs include those relating to drugs and medical supplies, healthcare personnel, travel, and losses in productivity of both sick and those caring for the sick [12,13]. In African regions, about 110,837 cases of cholera were estimated in 2007, resulting in an economic loss of US$ 43.3 million, US$ 60 million, and US$ 72.7 million, assuming life expectancies of 40, 53, and 73 years respectively [14]. The Zanzibar communities perceived cholera as a ‘fatal disease without treatment’ while fear of infecting others and isolation from others, obstruction with social relationships or daily activities, being sad, anxious, and worried were expressed as social and emotional impact of households [15]. Therefore, a comprehensive public-health package is needed to reduce the burden of disease. According to the World Health Organization (WHO), a comprehensive public-health package should consist of investment in safe water and sanitation, improved food safety, and the inclusion of a safe and affordable oral cholera vaccine to prevent cholera in high-risk populations [2,16-19]. However, implementing certain interventions in high-risk groups with poor knowledge of and attitudes toward cholera is not easy. Therefore, it is important to understand the current levels of knowledge, attitudes, and practices (KAP) of a given community to implement campaign programs, vaccination programs, and other preventive measures. Results of a study by Quick *et al*. suggest that acquiring behavioral information of a community is an important strategy in the control of cholera [20]. Results of a study in Tanzania indicate very poor prevention practices in spite of high levels of correct knowledge (85%) of and positive attitude (97%) toward cholera [21]. Another study identified that incorrect information, poor commitment of health staff to the community, and cultural factors were contributors to poor reporting of cholera cases [22]. The KAP of mothers and patients relating to immunization can influence the success, delay, or failure of immunization programs [23-28]. In Bangladesh, however, data on the KAP of households relating to cholera and the potential use of oral cholera vaccines are not available. We postulate that such data would help develop interventions to control cholera, especially in high-risk populations.

Health education is an important component of achieving national and international public- health goals, by encouraging the adoption of practices that benefit health. Thus, the aim of the present study was to assess the knowledge of, attitudes toward, and preventive practices relating to cholera and oral cholera vaccine among an urban population residing in a high cholera-prone setting in Dhaka, Bangladesh.

## Methods

### Study design

This cross-sectional study, conducted during December 2010–February 2011, used both quantitative and qualitative data-collection techniques.

### Study setting and population

The study area included 90 clusters (neighborhoods) in an area of high cholera incidence in Mirpur, with a target population of 240,000 [6]. Mirpur is the northwest part of capital Dhaka city of Bangladesh, the area of which has a mixed income neighborhood covering about 2.5 million people [6,29]. The 90 clusters were identified through an ongoing demographic surveillance system of “The introduction of cholera vaccine in Bangladesh (ICVB) project”. The project assigned the clusters randomly to three arms: one-third of the clusters were allocated as control, a third received only vaccine, and the remaining clusters received vaccine plus hygiene and behavior-change intervention messages [30,31]. The study population of the project included a population at high risk for cholera but excluded pregnant women and children aged less than one year. All adult men and women of the ICVB project were considered part of the KAP study population.

### Sampling and sample-size

Considering an estimate of 50% knowledge level (as the prevalence of knowledge of cholera in this community was unknown), with 5% precision of error, 95% confidence interval, and 80% power, the required sample-size was 384 for each arm. Taking into account a design effect of 2 and an estimate of 20% non-response rate, the sample-size was calculated to be 960 in each arm. Therefore, the total sample-size for three arms of the KAP survey was 2,880.

Since our study area included 90 clusters, we used cluster-sampling methodology for data-collection. In total, 15 clusters were randomly selected for the KAP study. Approximately 200 households from each cluster were randomly selected from the household list prepared by the demographic surveillance maintained by the ICVB project. An adult from each household who could provide information regarding his/her family was purposively selected for interview.

In-depth interviews were also conducted with 30 respondents from the selected clusters of the quantitative survey. The respondents were chosen purposively considering the characteristics, such as occupation, education, age, and sex (see Additional file 1).

### Data-collection

#### Survey

Experienced male and female interviewers collected data. The interviewers were trained by investigators, immunization experts, and public-health specialists. Both classroom and field training were imparted to the interviewers.

A semi structured questionnaire was used for collecting quantitative data (see Additional file 2). It comprised a series of questions developed through literature review [9,32,33] and revised to match with the cultural views. Multiple answers were recorded per question based on predefined categories but no probing of remaining unmentioned categories was done. The questionnaire was divided into the following three main sections:

**Knowledge on cholera and cholera vaccine** This section included 42 semi structured items on causes, management, treatment sources, and prevention measures of cholera, including knowledge on cholera vaccine, such as the number of doses, the dose interval, eligibility, and adverse effects. We also used one open-ended question in this section, “What do you understand by cholera/what is cholera”, to capture the respondent’s definition of cholera. We then entered the answers to this open-ended question, as structured responses, into the database.

**Practice relating to cholera and cholera prevention** This section included questions on care-seeking practices for the management of cholera (type of treatment, treatment places) and cholera-prevention practices.

**Attitudes toward cholera and cholera vaccine** This section included statements concerning the risks of cholera and cholera immunization (“cholera is very serious for adult or children”, “there may be side-effects of cholera vaccination”), perceived efficacy of various prevention measures (e.g. handwashing and proper sanitation practices), and the benefits of cholera immunization (“immunizations are effective in the prevention of disease”).

### Definitions

Based on the guidelines of cholera [34,35], the variables were defined as follows:

*Cholera:* Acute watery stool with or without vomiting.

*Causes of cholera:* Ingestion of food or water contaminated with *V. cholerae*. We divided this definition into three responses:

i. Lack of safe drinking-water or drinking contaminated water

ii. Eating rotten food/lack of food protection against contamination

iii. Infected by cholera germ (*V. cholerae*)

*Type of cholera management:* Oral rehydration salts (ORS), intravenous fluids, home-made saline solutions, lightly-salted rice water, or plain water.

*Places of cholera management:* Oral rehydration solution should be given early at home while cholera treatment centers or healthcare facilities should be used for proper clinical care. We considered two responses for knowledge scale here: home and outside home/treatment center.

*Cholera-prevention measures:* Provision of safe water and availability of proper sanitation and health education to the communities are prevention measures, including adherence to adequate food safety and to basic hygiene practices by individuals. This included five characteristics:

(i) Safe water: Use of boiled or tablet-treated or tubewell water for drinking and household work

(ii) Proper sanitation: Use of sanitary latrine/satisfactory sewage system/proper sanitary disposal of stool/feces

(iii) Health education: Attending health-education   sessions/health-education advice to drink   tubewell/ boiled/tablet-treated water, etc.

(iv) Food safety: Housefly-control measures/keeping food   covered/taking fresh food and avoiding rotten food

(v) Basic hygiene practices: Good-hygiene   practice/ washing hands with soap before   meal/ washing hands with soap after defecation

#### Qualitative data

Qualitative data were collected to supplement quantitative data. We recruited two experienced qualitative interviewers to conduct in-depth interviews using a guideline. Knowledge on cholera, including its causes, prevention, management and details of problems or barriers that the respondents faced in seeking healthcare were collected. The interviewers received training to ascertain the perceptions of respondents about cholera and cholera vaccination. All the interviews were conducted at the households of respondents during their leisure time. These interviews were conducted face to face lasting for 1-1½ hours, and digital audio recorders were used for recording the interviews.

### Analysis of data

#### Analysis of quantitative data

Data were entered into the visual BASICS/FoxPro software and analyzed using the SPSS software (version 11.5) (SPSS Inc., Chicago, IL). Sociodemographic variables of the respondents were collected from the demographic surveillance database of the ICVB project. Pearson’s Chi-square test was employed to determine the associations between the knowledge level of respondents and the sociodemographic, attitude and practice variables.

#### Measurement of knowledge

To measure knowledge of the respondents on cholera, a scoring system was used. Each correct response was scored as 1 while other responses, such as ‘incorrect’ or ‘don’t know’, were scored as 0 (zero). Since there were 16 variables (Table 1) in the knowledge section of the questionnaire, the total score ranged from 0 to 16. Using a frequency distribution [21], the poor knowledge was defined as a score of ≤8 (equal to or less than eight) while a score of ≥9 (equal to or greater than nine) was considered good knowledge.

**Table 1:** Respondents’ knowledge of cholera

**Characteristics**	**Given score**	**Percentage**
		**(n = 2,830)**
**Recognition of cholera**		
Watery stool with or without vomiting	1	23
**Causes of cholera**		
Lack of safe drinking-water, or drinking of polluted water	1	80
Eating rotten food/lack of food protection against contamination/if the food has not been covered up	1	83
Affected by cholera germ	1	1
**Type of cholera management**		
ORS	1	92
Rice saline	1	38
IV fluid	1	19
Home-made saline	1	38
Plain water	1	4
**Places of cholera management**		
Home	1	48
Health center	1	95
**Cholera-prevention measures**		
Use of safe water	1	74
Proper sanitation	1	7
Health education	1	7
Food safety	1	87
Basic hygiene practices	1	85
**Total given score**	16	
**Total mean score with SD**	7.79 ± 2.62	
**Good knowledge (score equal to or greater than 9)**		46 (95% CI: 43.9-47.5)
**Poor knowledge (score equal to or less than 8)**		54 (95% CI: 52.5-56.1)

#### Measurement of attitude

There were nine statements in the attitude section of the questionnaire giving a total score of 9 for the statements. The respondents were asked to indicate the extent of their agreement with the statements on whether they agreed or disagreed. A positive attitude was considered when a person agreed to a favorable outcome or disagreed with behavior, which has a negative impact on the prevention and control of cholera. Thus, a correct response (agreed to favorable outcomes or disagreed with negative behavior) about a statement was scored as plus one (+1) while a wrong response was scored as minus one (−1) [21]. If a respondent did not want to respond, or did not have any knowledge of a statement, he/she got a score of 0. Of the total score ranging from 0 to 9, a frequency distribution was carried out to find a cut-off point for positive or negative attitude. As the distribution indicated that most (99%) respondents were likely to have positive attitude toward cholera, we defined two groups: highly positive with a score of 5–9 and less positive with a score of 4 or less.

#### Scoring system to measure cholera-prevention practices

We used five items to measure the cholera-prevention practices, and a total of five points were assigned. Good practices were considered following a score of 3–5 and poor practices a score of 2 or below.

#### Predictors of a poor level of knowledge

Univariate and multivariate analyses were carried out to estimate unadjusted and adjusted odds ratios to identify the predictors of a poor level of knowledge.

Results of previous studies showed that a knowledge score was a significant predictor of attitude and practice scores [36,37]. We, thus, considered dichotomous variables and created ranks according to the response category of poor or good knowledge of cholera as the outcome variable [38]. The statistical significance of all results was considered when the p value was <0.05.

### Analysis of qualitative data

Qualitative information collected through in-depth interviews was transcribed and translated into English and analyzed using content analysis. The data-analysis process followed a sequence of interrelated steps, such as reading, coding, displaying, reduction, and interpretation. At first, the transcripts were carefully read, and then data were coded. Reading and coding were initiated while the data were collected. The data-display and reduction process was conducted at desk once all the data were collected. The inconsistencies of data (if any) were clarified through re-visit of field and reduction of non-standard data. Even during data display and reduction, the authors reviewed earlier steps to refine codes, reread texts, and revise some aspects of the analysis.

### Ethical consideration

All respondents gave written consent before participating in the study. The Research Review Committee and the Ethical Review Committee of icddr,b approved the study.

## Results

### Knowledge on cholera

In total, 2,830 of the 2,880 respondents were successfully interviewed (Table 1). The remaining households could not be interviewed even after several attempts because of absenteeism of respondents.

Table 1 shows the respondents’ knowledge on cholera. Only 23% of the respondents could correctly recognize cholera. Most respondents stated that eating unprotected or rotten food (83%) and drinking unsafe water (80%) were the main causes of cholera. About 92% of the respondents were aware of ORS. Regarding facilities where a patient can be treated, most (95%) mentioned outside home or treatment centers, followed by home (48%). Regarding the prevention measures of cholera, food safety, maintaining good-hygienic practices, use of safe water for drinking and household purposes, health-education activities were mentioned by 87%, 85%, 74%, and 7% of the respondents respectively. According to knowledge scoring, less than half of the respondents had good knowledge, and about 54% had poor knowledge of cholera.

All the respondents who were asked to participate in qualitative interviews complied. The qualitative data showed that the respondents could identify cholera patients through common symptoms, such as acute loose motion and vomiting. Most respondents (27 of 30) who participated in in-depth interviews stated that a cholera patient might have the following symptoms: dehydration, loss of appetite, lethargic, unconsciousness, hypothermia, anorexia, unable to eat, vertigo, loss of energy, slackening of the body, or abdominal pain. A few respondents (7 of 30) could differentiate diarrhea from cholera. They perceived that diarrhea is a mild condition while cholera is the severe form. The respondents stated that when diarrhea became severe, a patient became restless within 3–4 hours of being affected by diarrhea and needed to be hospitalized; this condition could be then termed as cholera.

Most in-depth interview participants (28 of 30) also mentioned that eating unhygienic or rotten food and drinking contaminated water, or water, which was not boiled or filtered, were the causes of cholera.

A few participants (3 of 30) believed that being affected by cholera is God’s will. Another three respondents mentioned that cholera was caused by a cholera germ. One of them said:

“When any member of a household is affected by cholera, other members should be careful. Otherwise, the germ from the affected person will spread to others.”

Results of analysis of qualitative data also showed that there were three patterns of responses regarding knowledge on cholera treatment. These included: home management, treatment at nearby drug stores or private clinics, and tertiary-level healthcare facilities. They stated that ORS could be bought from local pharmacies to start treating cholera, and anybody could administer this treatment to the cholera patient at home.

Most in-depth interview participants (29 of 30) mentioned various preventive measures for cholera. These included cleanliness of living places and surroundings; having hygienic food; use of safe water for drinking and domestic purposes; handwashing; and use of sanitary latrines.

### Knowledge on cholera vaccine

The findings revealed that only 16% of the participants had heard of cholera vaccines.

Qualitative data revealed that a few participants (9 of 30) had heard of cholera vaccine but did not have in-depth knowledge. They knew that this vaccine is available from non-governmental organizations (NGOs). One respondent said that he received two doses of a cholera vaccine in childhood through injection.

### Attitudes toward cholera and oral cholera vaccine

Attitudes toward cholera and oral cholera vaccines are presented in Table 2. The largest proportion of the respondents showed a positive attitude toward cholera and oral cholera vaccines, with a high level of agreement for different statements, such as “washing both the hands with soap or ash after defecation”, “defecating in an open place could lead to disease”, “washing hands before taking food”, “cholera is a severe health problem which may cause death”, and “cholera is very serious for adults and children”. About 86% of the respondents agreed that cholera could be prevented through vaccination. About two-thirds disagreed with the statement that the cholera vaccine might be harmful for health. The attitude scale showed that 97% of the study participants had a highly positive attitude toward cholera and oral cholera vaccine.

**Table 2:** Attitude toward cholera and cholera vaccine (n = 2,830)

**Statement**	**Agreed**		**Disagreed**	
	**Given score**	**Percentage**	**Given score**	**Percentage**
Believe that we should wash our both hands with soap or ash after defecation	+1	99.9	−1	0.1
Believe that it may cause disease if stool is passed at anywhere	+1	99.9	−1	0.1
Believe that we should wash our hands before taking any food	+1	100.0	−1	0
Believe that we should encourage people for cholera vaccination	+1	99.9	−1	0.1
Believe that cholera is very serious for children	+1	98.2	−1	1.8
Believe that cholera is a very serious disease for adults	+1	97.5	−1	2.5
Believe that cholera is a severe health problem which may cause death	+1	99.5	−1	0.5
Believe that cholera can be prevented through vaccination	+1	86.4	−1	0.8, *don’t know- 12.8
Believe that cholera vaccine may be harmful for health	−1	2.4	+1	64.4, *don’t know- 33.3
**Total given score**	9			
**Mean score of attitude with SD**	7.14 ± 0.80			
**% of highly positive (score equal to or greater than 5)**	97.0 (95% CI: 96.4-97.6)			
**% of less positive (score equal to or less than 4)**	3.0 (95% CI: 2.4-3.6)			

* Don’t know responses were scored as 0.

### Practices relating to prevention of cholera

#### Cholera prevention

Table 3 presents the respondents’ practices relating to cholera-prevention measures. About 89% of the respondents practiced food safety by having fresh food and avoiding rotten food and maintained good-hygiene (86%) to prevent cholera in their households. Two-thirds of the respondents used safe water for drinking and household purposes. Eight percent attended health-education sessions, and 7% maintained proper sanitation practices. Overall, 61% followed good practices to prevent cholera.

**Table 3:** Practices of respondents relating to prevention of cholera

**Characteristics**	**Given score**	**Percentage**
		**(n = 2,830)**
**Cholera-prevention measures**		
Use of safe water	1	67.7
Proper sanitation	1	7.3
Health education	1	8.2
Food safety	1	88.9
Basic hygiene practices	1	86.2
**Total given score**	5	
**Total mean score with SD**	2.58 ± 0.85	
**Good practice (score equal to or greater than 3)**		60.6 (95% CI: 58.8-62.4)
**Poor practice (score equal to or less than 2)**		39.4 (95% CI: 37.6-41.2)

#### Care-seeking for cholera

About 6% (n = 182) of the respondents reported that someone in their households suffered from cholera in the last six months before data-collection. About 94% (n =182) of the cholera patients were confirmed by pathological tests. The patients sought treatment at home (46%) and/or visited local pharmacies (24%). Others visited the icddr,b hospital (data not shown).

#### Barriers faced in treatment or prevention practices

Qualitative data showed that the financial problem was the main barrier to seeking treatment by cholera patients. Over one-third of the respondents (11 of 30) who participated in in-depth interviews said that they were too poor to visit a doctor, buy medicines, and/or organize transportation to a hospital. However, a few respondents stated that they had received free treatment from the icddr,b hospital but they had to spend money for travel and food. They also stated that bad traffic was the cause for delayed arrival at the hospital.

Nearly half of the respondents (14 of 30) stated that they were aware of the effects of cholera but they could not always drink boiled water as they did not have adequate water, gas, and fuel supply. Besides, they could not take fresh food as they could cook only once a day, or they had to cook food in their neighbor’s house in a common kitchen. Inadequate drainage systems were also mentioned by the respondents as a factor leading to cholera susceptibility.

### Association of knowledge with sociodemographic, attitudinal and practice-related characteristics

Table 4 shows the association of the respondents’ knowledge on cholera with the sociodemographic, attitude, and practice-related characteristics. Respondents with poor knowledge of cholera were seen in all age-groups with no significant differences. On the other hand, males (59%) were more likely to have poor knowledge than females (52%) (p = 0.001). Respondents with secondary and higher levels of education (48%) were significantly less likely to have poor knowledge compared to those with primary (55%) or having no education (58%) (p < 0.001). Independent earners (59%) were more likely to have poor knowledge on cholera than nonworking (51%) and nonindependent earners (53%). Respondents who had a monthly overall household expenditure of US$ 88 or below (58%) were more likely to have poor knowledge compared to respondents with higher monthly overall expenditure. In both the cases (occupation and monthly expenditure), the difference was significant (p < 0.05). Although the number of rooms per household and family-size did not show any significant association with the respondent’s knowledge level, findings showed that more than half of the respondents with poor knowledge were living in only one room with a family of 4 or less members (55%).

**Table 4:** Association of knowledge of respondents on cholera with sociodemographic, attitudinal and practice-related characteristics

**Characteristics**	**Knowledge level**		**Total**	**p value**
	**Poor knowledge (%)**	**Good knowledge (%)**	**% (n)**	
Status of knowledge	54.3 (n = 1,538)	45.7 (n = 1,292)	100.0 (2,830)	
**a. Sociodemographic characteristics**				
**Age-group (years)**				
18-23	55.2	44.8	18.9 (536)	0.086
24-34	56.9	43.1	36.0 (1020)	
35-44	50.8	49.2	25.1 (711)	
45+	53.5	46.5	19.9 (563)	
**Sex**				
Male	59.4	40.6	28.3 (800)	0.001
Female	52.4	47.6	71.7 (2030)	
**Education**				
No education	57.9	42.1	47.1 (1332)	0.000
Primary education	54.7	45.3	25.7 (726)	
Secondary and higher	47.9	52.1	27.3 (772)	
**Occupation**				
Nonworking respondents^a^	51.0	49.0	44.1 (1249)	0.001
Nonindependent in profession^b^	53.3	46.7	34.91 (593)	
Independent earners^c^	59.2	40.8	20.99 (988)	
**Monthly expenditure in taka**^**d**^				
≤7000 (≤88 US$)	57.6	42.4	34.3 (969)	0.006
7001-9600 (88.1-120 US$)	55.0	45.0	32.4 (916)	
>9600 (>120US$)	50.4	49.6	32.4 (940)	
**Family-size**				
≤ 4	55.3	44.7	57.7 (1632)	0.133
5 and more	53.1	46.9	42.3 (1198)	
**No of living rooms**				
1	54.8	45.2	83.7 (2369)	0.153
1+	52.1	47.9	16.3 (461)	
**b. Attitudinal characteristics**				
**Positive attitude toward cholera, its prevention and cholera vaccine**				
Highly positive (score equal to or greater than 5)	53.4	46.6	97.0 (2745)	0.000
Less positive (score equal to or less than 4)	84.7	15.3	3.0 (85)	
**c. Practice-related characteristics**				
**Cholera-prevention practices**				
Good practice (score equal to or greater than 3)	39.0	61.0	60.6 (1714)	0.000
Poor practice (score equal to or less than 2)	77.9	22.1	39.4 (1116)	

^a^Nonworking respondents included housewives or unemployed women who spent time at home and had no income.

^b^Nonindependent in profession includes: mainly involved in different organizations with or without fixed compensation, such as service holders, teachers, pensioners, and students).

^c^Independent earners are those who have their own control to their work and spend time according to their wish (business, daily wage earners, transport workers).

^d^Overall monthly expenditure of a household has been categorized based on terciles.

Respondents who had a less positive attitude toward cholera (85%) were significantly more likely to have poor knowledge compared to those with a highly positive attitude (53%). Likewise, respondents maintaining poor prevention practices (78%) were more likely to have poor knowledge compared to those with good practices (39%) (p < 0.001).

### Predictors of poor level of knowledge on cholera

Table 5 presents the results of logistic regression analyses of the respondents’ knowledge on cholera as the only outcome variable with different independent variables, such as sociodemographic characteristics, attitudes, and prevention practices. Sex, education, occupation, monthly household expenditure, positive attitude, and cholera-prevention practices were associated with the outcome variable through Chi-square tests. Therefore, these variables were included to measure unadjusted odds in a regression model.

**Table 5:** Results of logistic regression analyses of respondents’ knowledge on cholera by sociodemographic, attitudinal and practice-related characteristics

**Characteristics**	**Models**	
	**Unadjusted OR (95% CI)**	**Adjusted OR (95% CI)**
**Sociodemographic**		
**Sex**		
Male	1.00	1.00
Female	0.75 (0.64-0.89)**	0.74 (0.62-0.87)*
**Education**		
No education	1.49 (1.25-1.78)**	
Primary education	1.31 (1.07-1.61)*	
Secondary and higher	1.00	
**Occupation**		
Nonworking respondents	0.91 (0.75-1.11)	
Independent earners	1.27 (1.03-1.56)*	
Nonindependent earners	1.00	
**Monthly expenditure in taka**		
≤7000 (≤88 US$)	1.33 (1.11-1.59)*	1.31 (1.09-1.58)*
7001-9600 (88.1-120 US$)	1.20 (1.00-1.44)*	1.19 (0.99-1.43)
>9600 (>120 US$)	1.00	1.00
**Positive attitude toward cholera, its prevention and cholera vaccine**		
Less positive (score equal to or greater than 5)	4.83 (2.66-8.76)**	4.92 (2.71-8.94)**
Highly positive (score equal to or less than 4)	1.00	1.00
**Cholera-prevention practices**		
Poor practice	5.49 (4.63-6.52)**	
Good practice	1.00	

*p < 0.05; **p < 0.001.

Although unadjusted models showed that education (x), occupation (y), and monthly expenditure (z) were significantly associated with the knowledge level of the respondents, they highly correlated (r_xy_ = −0.079, r_yz_ = 0.139, r_xz_ = 0.128. These models also showed that the associations between knowledge and monthly expenditure and the association between knowledge and education were better than the association between knowledge and occupation. Given the relationship between education and knowledge, we included monthly household expenditure in the adjusted model.

The respondents’ attitudes and prevention practices also significantly correlated (r = 0.061 p < 0.01). Since both the variables were highly associated with the knowledge level, we selected one variable (respondent's attitude) purposively for the final model. Finally, we adjusted the respondents’ sex, monthly expenditure, and positive attitude in the full model, and all the three variables were significant.

Females were less likely to have poor knowledge compared to males [odds ratio (OR): 0.74; 95% confidence interval (CI): 0.62-0.87]. Respondents who had the lowest monthly household expenditure (≤ US$ 88) were more likely to have poor knowledge compared to respondents who had high monthly household expenditure (>US$ 120) (OR: 1.31; 95% CI: 1.09-1.58). Respondents with a less positive attitude were more likely to have poor knowledge compared to those with a high positive attitude (OR: 4.92; 95% CI: 2.71-8.94).

## Discussion

Few studies have explored the community perceptions and practices relating to diarrheal diseases in Bangladesh [39,40]. The present study assessed the knowledge of, attitudes toward, and prevention practices relating to cholera and oral cholera vaccination among urban slum-dwellers. We found that only 23% of the respondents could identify cholera as watery diarrhea, which was lower than that observed in studies in Zanzibar and Haiti where 60-89% of the population could characterize cholera as watery stool or diarrhea [15,41,42]. Although the definition of cholera varies even in the WHO documents, we used a commonly-used term as a correct response on defining cholera, i.e. ‘acute watery stool with or without vomiting’ [34] and gave a ‘zero’ score if a respondent could not specify diarrhoea as watery, which may result in a low recognition of cholera in our study. Conversely, the Haiti study commonly identified cholera as diarrhea (89.1%), and the Zanzibar study presented to respondents a clinical vignette describing a cholera case and asked how they would name the condition presented to them. Results of a community-based study showed that cholera was the result of diarrhoea, vomiting, dehydration, and weakness of the body, which corroborated the qualitative findings of our study [22]. Despite the availability of oral cholera vaccine through NGOs, it has not been incorporated in Bangladesh’s Expanded Programme on Immunization (EPI) schedule, which may be the reason behind the poor knowledge of the respondents on oral cholera vaccine.

The use of ORS in the management of cholera is widely known [22,41]. We obtained findings similar to the findings of a study in Haiti where more than 90% of respondents had knowledge of ORS [42]. A Zanzibar study reported that the peri-urban people are more likely to give ORS to patients primarily for the treatment of cholera than the rural people [15]. Results of another study showed that the use of antibiotics, in addition to ORS, is very common in Bangladesh [43].

Less than 10% of the respondents of the present study knew that proper sanitation and health education are important preventive measures. This may be because of the poor infrastructure of urban slum sanitation [44] and lack of health-education activities in the community. It was observed during data-collection that some NGOs were providing health education in some areas, and only the respondents of those areas would mention health education as a preventive measure. Overall, 54% of the respondents had poor knowledge on cholera. This result differed from those of other studies where respondents had a high level of knowledge on cholera [21,45,46].

The findings of the present study revealed that the respondents had poor knowledge but the level of positive attitude toward cholera and cholera vaccine was very high. The success of the EPI in Bangladesh could be the reason for delivering a positive attitude toward any vaccine among Bangladeshi people. However, the attitude level found was similar to the findings of a study in Tanzania [21]. On the other hand, in Bangladesh, the term’cholera’ is not reported in newspapers and other media accessible to the population, and epidemics of cholera are reported as ‘diarrhea’ which can be the possible explanation of discrepancy between the level of knowledge and attitude among the study participants.

Although results of other studies showed that practice lagged behind knowledge [20,22], the results of this study showed that the respondents with good knowledge about cholera followed better prevention practices. This may be due to a response bias, such that the respondents who had knowledge on cholera and its prevention may conceal their actual practices. Inclusion of observation methods as part of data-collection may validate this information, which is a limitation of this study. On the other hand, the participants taking part in the in-depth interviews stated that they could not maintain preventive measures for cholera because of the poor infrastructure in their household dwellings in the slums.

In Bangladesh, pharmacies and drug-sellers are the firstline and dominant healthcare providers [47,48], which support our findings. The in-depth interview participants reported that lack of financial means was the main constraint for seeking treatment, followed by transportation costs to reach a treatment center. To clarify this pattern, a further study on the financial burden of cholera on households should be considered.

We found a significant association between the respondents’ knowledge and several factors, including sex, education, occupation, monthly overall household expenditure, a positive attitude toward cholera, and practices to prevent cholera. Education is primarily important and is related to knowledge. Female and nonworking respondents were more knowledgeable as they were the main caregivers of cholera patients, which is consistent with results of a study [22]. Independent earners were 27% more likely to have poor knowledge than nonindependent earners, likely due to differences in education. Similarly, those who spend a lower amount of money for their households each month may not have much access to healthcare; therefore, they are more likely to be unaware of the disease. In the adjusted model, males, lower monthly expenditure (≤88 US$) and a lower positive attitude toward cholera were the significant predictors of having poor knowledge.

Based on the results of these analyses, a few recommendations may be made. The findings revealed that the slum-dwellers had partial knowledge on cholera and cholera vaccine-related issues. Therefore, undertaking a health-education intervention program is essential to educate and motivate people to prevent and control cholera. The target groups for such an educational program should be those who are illiterate or with lower formal education, males, independent earners who have lower monthly household expenditures, and those with a lower positive attitude toward cholera and cholera vaccine. They should also be taught on the use of water-treatment procedures. Information on the location of nearby health centers where treatment of cholera is available should also be disseminated to the people. Finally, many slum-dwellers reported that they could not maintain good hygiene practices to prevent cholera due to a scarcity of water, gas supply, and the proper sanitation and drainage system. In the slums of Bangladesh, for many reasons, ensuring these to this community is very difficult and will take a long time. Therefore, the introduction of cholera vaccination among the slum population would be an important step to prevent and control cholera. Perceptions of the target population about vaccine and their willingness to be vaccinated before introducing such a programme should also be considered for successful implementation.

## Conclusion

This study was conducted with a group of people who are at high risk of cholera [6], and their knowledge of, attitude toward, and prevention practices relating to cholera and oral cholera vaccination were analyzed. The findings revealed a poor level of knowledge on cholera among this high-risk group. Strengthening of health-education activities may, thus, improve their knowledge on cholera. Such education should focus on cholera prevention and control, including detailed information on cholera vaccine (e.g. vaccine availability, sources of vaccine, etc.). The data of the present study are also important to implement future vaccination campaigns for population at high risk to prevent and control cholera.

## Competing interests

The authors declare that they have no competing interests.

## Authors’ contributions

JU, FQ, IAK, TW, AS, and AC were involved in developing concept, study implementation, data analyses, and writing the manuscript. SSTK, TW, and JU collected and analyzed the qualitative data. NCS and AUS had inputs in designing quantitative sampling technique and analysis of data. FK, FC, AS, and AIK also interpreted the medical terminologies and gave technical support. All the authors read and approved the final manuscript.

## Pre-publication history

The pre-publication history for this paper can be accessed here:

http://www.biomedcentral.com/1471-2458/13/242/prepub

## Supplementary Material

Additional file 1 **Selective criteria of in-depth interview participants.** An additional text file has been included as a table which shows the demographic and detailed information relating to selection of a respondent for in-depth interviews in the field. Click here for file

Additional file 2 **Questionnaire for KAP household survey.** The questionnaire that we have used for collection of quantitative data has been attached as an additional file 2. Click here for file
