# Supplementary material for: Knowledge of, attitudes toward, and preventive practices relating to cholera and oral cholera vaccine among urban high-risk groups: findings of a cross-sectional study in Dhaka, Bangladesh
Source: BMC Public Health. 2013 Mar 19;13:242. doi: 10.1186/1471-2458-13-242 (PMC3608226; doi:10.1186/1471-2458-13-242)
Supplement: Additional file 1 — Selective criteria of in-depth interview participants. An additional text file has been included as a table which shows the demographic and detailed information relating to selection of a respondent for in-depth interviews in the field. [file 1471-2458-13-242-S1.doc]

**Additional file 1: Selective criteria of in-depth interview participants**

| **Occupation** | **Education group** | **Age group** | | **Sex** | | **Total** |
| --- | --- | --- | --- | --- | --- | --- |
|  |  |  |  | **Male** | **Female** |  |
| Housewife | Primary | 25-34 |  | | 3 | 3 |
|  | Secondary | >=24 |  | | 3 | 3 |
|  |  | 25-34 |  | | 2 | 2 |
|  |  | 34+ |  | | 2 | 2 |
|  | Secondary plus | 34+ |  | | 3 | 3 |
| Service | Primary | >=24 | 0 | | 1 | 1 |
|  |  | 25-34 | 1 | | 0 | 1 |
|  | Secondary | 25-34 | 1 | |  | 1 |
|  | Secondary plus | 25-34 | 1 | |  | 1 |
| Transport worker | Primary | 34+ | 2 | |  | 2 |
|  | Secondary | 25-34 | 1 | |  | 1 |
|  | Secondary plus | 25-34 | 1 | |  | 1 |
|  |  | 34+ | 3 | |  | 3 |
| Business | Secondary | 25-34 |  | | 1 | 1 |
|  | Secondary plus | 25-34 | 1 | |  | 1 |
|  |  | 34+ | 2 | |  | 2 |
| Student | Secondary plus | >=24 | 2 | |  | 2 |
| Total |  |  | 15 | | 15 | 30 |
