# Supplementary material for: Knowledge of, attitudes toward, and preventive practices relating to cholera and oral cholera vaccine among urban high-risk groups: findings of a cross-sectional study in Dhaka, Bangladesh
Source: BMC Public Health. 2013 Mar 19;13:242. doi: 10.1186/1471-2458-13-242 (PMC3608226; doi:10.1186/1471-2458-13-242)
Supplement: Additional file 2 — Questionnaire for KAP household survey. The questionnaire that we have used for collection of quantitative data has been attached as an additional file 2. [file 1471-2458-13-242-S2.pdf]

International Centre for Diarrhoeal Disease Research, Bangladesh
Questionnaire for KAP household survey

Household ID [| | | | | | |] Name of respondent [ ]

GIS ID [| | | | |] Name of household head [ ]

Household serial of respondent [|] PID [| | | | | | | | | |]

**Section 1: Identification & Eligibility of respondent (Please, conduct the interview if age=>18 years)**				
Q. No	Questions	Response Categories	Code	Skip Code
101	Are you a member of this family?	Yes ....1<br>No 2	→	Carry on the interview
102	Are you pregnant now?	Yes ....1<br>No ....2<br>Not applicable..............................3	→	End the interview
103	Interview result	Complete ....1<br>Incomplete...................................2		
104	Name of interviewer _______________ ID [ ] Date [					]				
105	Name of supervisor _______________ ID [ ] Date [					]				
106	Name of the data entry person ____________ ID [ ] Date [					]				

**Section2: Information about household assets**				
**Q. No**	**Questions**	**Response Categories**	**Code**	**Direction**
201	Family assets	Yes............1 No............2		
	a. Television	Yes............1 No............2		
	b. Radio	Yes............1 No............2		
	c. Cassette player	Yes............1 No............2		
	d. DVD/CD player	Yes............1 No............2		
	e. Bicycle	Yes............1 No............2		
	f. Motorcycle	Yes............1 No............2		
	g. Chair / Sofa	Yes............1 No............2		
	h. Table	Yes............1 No............2		
	i. Sewing machine	Yes............1 No............2		
	j. Mosquito net	Yes............1 No............2		
	k. Electricity	Yes............1 No............2		
	l. Gas supply	Yes............1 No............2		
	m. Almirrah /wardrobe / showcase	Yes............1 No............2		
	n. Bed	Yes............1 No............2		
	o. Mobile phone	Yes............1 No............2		
	p. Land phone	Yes............1 No............2		
	q. Refrigerator	Yes............1 No............2		

Section 3: Knowledge on cholera and cholera vaccine

Section 3a: Knowledge on cholera, its prevention and treatment (please, avoid leading question)				
**Q. No**	**Questions**	**Response Categories**	**Code**	**Direction**
301	Do you know name of any waterborne diseases.	Yes ....1<br>No 2	→	303
302	If yes, what are these?	Diarrhoea/ Cholera....................A<br>Cholera....................B<br>Dysentery……………………C<br>Hepatitis....................D<br>Typhoid ....E<br>Others....................F<br>Do not know....................G	→<br>→	303a-305<br>306a
303	Have you ever heard of diarrhoea?	Yes ....1<br>No 2	→	306
303a	What do you mean by diarrhoea?	________________<br>________________<br>________________		
304	What were the signs & symptoms of diarrhoea?	Passage of 3 or more loose or water stool in 24 hours……………..............A<br>Repeated vomiting …....................B<br>Severe dehydration within very short time ....C<br>Muscles cramp....................D<br>Scanty Urination....................E<br>Other....................F		
305	What are the causes of diarrhoea?	Lack of safe drinking water ....A<br>Inadequate of safe water supply system............ B<br>Unhygienic disposal of excreta and refuse .... C<br>High density of population ....D<br>Hot humid climate. ....E<br>Illiteracy, ignorance, and lack of health education……..………………..…………….....F<br>Eating rotten food, lack of food protection against contamination...…………...…….…..….G<br>Touching food without cleaning hand by soap….....................H<br>Not washing hands after defection …..................I<br>Unhygienic living environment……….……......J<br>Not taking vaccines....................K<br>Affected by cholera germ ....L<br>Affected by Rota virus ....M<br>Others.........................…………...N<br>Do not know ....O		.
306	Have you ever heard of cholera?	Yes ....1<br>No 2	→	319
306a	What do you mean by cholera?	________________<br>________________<br>________________		

Q. No	Questions	Response Categories	Code	Direction
307	What are the signs & symptoms of cholera?	Passage of 3 or more loose or water stool in 24 hours…………………………………….............A<br>Repeated vomiting …..B<br>Severe dehydration within very short time ....C<br>Muscles cramp...D<br>Scanty Urination..E<br>Other...F	→	If the response is 307c, then go to 308, otherwise, skip to 311
308	Do you know how to recognize the several stages of dehydration?			
308a	No symptom of dehydration<br>(Multiple answers acceptable)	Condition well, alert……..................................A<br>Eyes are normal.......................……………...B<br>Presence of tears in eyes...................................C<br>Mouth and tongue are moist…...........................D<br>Not thirsty, drink normally...............................E<br>Skin pinch goes back very quickly………….. F<br>Others.......................................…………G<br>Do not know ....H		
308b	Some symptoms of dehydration<br>(Multiple answers acceptable)	Condition Restless, irritable.............................A<br>Eyes are sunken.......................……………B<br>Absence of tears in the eyes.............................C<br>Mouth and tongue are dry ....D<br>Thirsty, drink water eagerly..............................E<br>Skin pinch goes back slowly………..………..F<br>Others...……..…G<br>Do not know ....H		
308c	Severe dehydration<br>(Multiple answers acceptable)	Lethargic or unconscious, floppy ....A<br>Eyes are very sunken & dry.............................B<br>Absence of tears in the eyes.............................C<br>Mouth and tongue are very dry.........................D<br>Drink poorly or unable to drink……..................E<br>Skin pinch goes back very slowly..................…..F<br>Others.......................................…………G<br>Do not know ....H		
309	What would be the consequences of dehydration?	Unconsciousness due to severe dehydration<br>……………………………………….............A<br>Kidney failure ....B<br>Death...C<br>Others....................................……………D<br>Do not know ....E		
310	In which stage of dehydration, should a patient be taken to Hospital?	No symptom of dehydration............................1<br>Some symptoms of dehydration.......................2<br>Severe dehydration..3		

Q. No	Questions	Response Categories	Code	Direction
311	What are the causes of cholera?<br>(Multiple answers acceptable)	Lack of safe drinking water ....A<br>Inadequate of safe water supply system............ B<br>Unhygienic disposal of excreta and refuse .... C<br>High density of population ....D<br>Hot humid climate. ....E<br>Illiteracy, ignorance, and lack of health education……..……………..……………....F<br>Eating rotten food, lack of food protection against contamination...………....……..……G<br>Touching food without cleaning hand by soap…..H<br>Not washing hands after defection …..................I<br>Unhygienic living environment………..……....J<br>Not taking vaccines...K<br>Affected by cholera germ ....L<br>Affected by Rota virus ....M<br>Others.......................................……………...N<br>Do not know ....O		
312	Do you know when does Cholera outbreak occurred each year?<br>(Multiple answers acceptable)	In Summer..A<br>In Rainy season ....B<br>In Winter ....C<br>Other……..……………………………………D<br>Do not know ....E		
313	What type of treatment should be given to a cholera patient?<br>(Multiple answers acceptable)	Packet saline ....A<br>Rice saline...B<br>IV saline ....C<br>Home-made saline..D<br>Zink tablet or syrup ....E<br>Antibiotics...F<br>Herbs..G<br>Homeopath...H<br>Plain water..I<br>Nothing ....J<br>Others...K<br>Do not know L		
314	Who can provide treatment for cholera?<br>(Multiple answers acceptable)	M.B.B.S Doctor……………………………….A<br>Nurse…………………………………………B<br>Family Welfare Visitor……..............................C<br>SACMO/Medical Assistant……………………D<br>Heath Assistant…………………………..…..E<br>Family Welfare Assistant…………………...…..F<br>Paramedics……………………………..…..G<br>ICVB Staff/Volunteer ………………………..H<br>Community Health Worker (NGO)………. ….I<br>Pharmacist/Drug sellers/Village Doctor …………………………………….…………...J<br>Kabiraj/Hekimi…………………………...…...K<br>Fakir……………………………………….....L<br>Homeopath Doctor………………….............M<br>Relatives……………………………………...N<br>Family Members……………………….…..O<br>Self……………………………………….…P<br>Neighbor……………………………………Q<br>Others (specify)………………………………..R<br>Do not know...................………….………...S		

Q. No	Questions	Response Categories	Code	Direction
315	Where can cholera treatment be provided?<br>(Multiple answers acceptable)	At home..A<br>Health centre..B<br>Other (please, specify) ....C<br>Do not know...D		
316	Do you know any health facility where treatment for cholera patient is provided?<br>(Multiple answers acceptable)	Mohakhali cholera Hospital, icddr,b.…………A<br>Mirpur cholera Hospital, icddr,b ………………B<br>Medical College Hospital (Public)……….…...C<br>Other Govt. hospital …………………………..D<br>Private clinic or hospital ....E<br>NGO clinic or hospital……………….……......F<br>Doctor's chamber...G<br>Allopathic Pharmacy ....H<br>Homeopath pharmacy ....I<br>Indigenous/ herbal treatment ....J<br>Herbal ....K<br>Others ....L<br>Don't know……………………………………M		
317	What do you know about the prevention measures of cholera?<br>(Multiple answers acceptable)	Use of safe water for drinking & household works...A<br>Provision of safe drinking water supply ....B<br>Use of sanitary latrine ....C<br>Satisfactory sewage system/ proper sanitary disposal of excreta, refuse.................................D<br>Washing hands with soap before meal...............E<br>Washing hands with soap after defecation.........F<br>Protection of foods (taking fresh food and avoiding rotten food) ....G<br>Housefly control measures............................... H<br>Early case finding in the community for the initiation of prompt treatment.......................... .I<br>Disinfection of all the contaminated articles by phenol, boiling or lime.....................................J<br>Good hygiene practice .... K<br>Health education (advises to drink tube well/ boiled/ tablet treated water etc.).......L<br>Taking cholera vaccine ....M<br>Others.. N<br>Do not know ....O		

Q. No	Questions	Response Categories		Code	Direction
318	How did you know about the cholera, its prevention and treatment?<br><br>(Multiple answers acceptable)	**a.Through ICVB**	**b.Other (excluding ICVB)**		
		Individual cards............... A<br>Mobile message .... B<br>Leaflet .... C<br>Media communication (print .... D<br>Media communication (electronic ....E<br>Inter personal communication (IPC) by house to house visit of health care providers........F<br>IPC by house to house visit of volunteers .... G<br>Banner............................. H<br>Others................................I<br>Not applicable .... J	Leaflet............................. A<br>Posters..............................B<br>Miking ....C<br>IPC with formal health care providers...... D<br>IPC with other CHWs/ volunteers..........................E<br>IPC with neighbour..........F<br>IPC with family/relatives G<br>Media communication (print)............................ H<br>Media communication (electronic) ....I<br>Street drama..................... J<br>Personal Experience ...... K<br>Others..............................L<br>Not applicable................M		
	I. How do you know about cholera? (Multiple answers acceptable)				
	II. How do you know about prevention? (Multiple answers acceptable)				
	III. How do you know about treatment? (Multiple answers acceptable)				
319	Do you know the names of vaccine preventable diseases?	Yes...1<br>No...2		→	321
320	If yes, what are those? (Multiple answers acceptable)	Tuberculosis..A<br>Diphtheria...B<br>Whopping cough.......................................C<br>Tetanus...D<br>Hepatitis...E<br>Pneumonia...F<br>Polio militias ....G<br>Measles, mumps, rubella ....H<br>Chickenpox...I<br>Cholera ....J<br>Other...K		→ (Cholera)	322
321	Have you ever heard about Cholera Vaccine?	Yes ....1<br>No...2		→ (No)	401
321a	If yes, what is the name of cholera vaccine?	ShanChol...1<br>Dukoral...2<br>Other...7<br>Don't know..9			

Q. No	Questions	Response Categories	Code	Direction
322	Do you know about required number of doses of Cholera vaccine?	....(Nos.)<br>Do not know 99	→	324
323	What is the interval between two doses?	....(Days)<br>Do not know..99<br>Not applicable…...………………………88		
324	Do you know how to administer the cholera vaccine?	Oral vaccine...1<br>Through injection...2<br>Other...7<br>Don't know...9		
325	Do you know who the eligible for cholera vaccine are? (Multiple answers acceptable)	Pregnant women...A<br>Less than one year children...............................B<br>One and above year age persons……………...C<br>Other...D<br>Don't know...E		
326	Do you know the sources of cholera vaccine? (Multiple answers acceptable)	Mohakhali cholera Hospital, icddr,b.………….A<br>Mirpur cholera Hospital, icddr,b ………………B<br>Medical College Hospital (public) ....C<br>Other Government hospital ....D<br>Private clinic/hospital.......................................E<br>NGO clinic/hospital..F<br>Private chamber ....G<br>Allopathic pharmacy...H<br>Homeopathic pharmacy......................................I<br>EPI centre…………………………………...…J<br>It is not available………………………………K<br>Other...L<br>Don't know…………….………….....………M		
327	Do you know about the benefits of cholera vaccine? (Multiple answers acceptable)	a. ________________________________<br>b. ________________________________<br>c. ________________________________		
328	Do you know any side-effect/ health problems of cholera vaccine? (Multiple answers acceptable)	Headache ....A<br>Itching ....B<br>Weakness ....C<br>Joint pain..D<br>Vomiting ....E<br>Diarrhoea ....F<br>low blood pressure ....G<br>Others ....H<br>Don't know ....I<br>No problem.………………………...…………..J	 <br><br><br><br><br><br><br>→<br>→	 <br><br><br><br><br><br><br>330<br>330

Q. No	Questions	Response Categories	Code	Direction
329	Do you know where should seek treatment for those problems/ side-effect?<br>(Multiple answers acceptable)	Mohakhali cholera Hospital, icddr,b.............A<br>Mirpur cholera Hospital, icddr,b ....B<br>Medical College Hospital (public) ....C<br>Other Government hospital ....D<br>Private clinic/hospital..E<br>NGO clinic/hospital...F<br>Private chamber ....G<br>Allopathic pharmacy..H<br>Homeopathic pharmacy.......................................I<br>EPI centre...J<br>Other...K<br>Don't know...L		
330	Do you know the price of cholera vaccine?	___________________ taka<br>Don't know...99		
331	How did you know about the cholera vaccine?<br><br>(Multiple answers acceptable)	**a. Through ICVB**<br>Individual cards.............. A<br>Mobile message .... B<br>Leaflet .... C<br>Media communication (print............................... D<br>Media communication (electronic ....E<br>Inter personal communication (IPC) by house to house visit of health care providers ....F<br>IPC by house to house visit of volunteers.................... G<br>Banner .... H<br>Others................................I<br>Not applicable .... J<br><br>**b. Other (excluding ICVB)**<br>Leaflet .... A<br>Posters.............................B<br>Miking ....C<br>IPC with formal health care providers...... D<br>IPC with other CHWs/ volunteers.........................E<br>IPC with neighbour.......... F<br>IPC with family/relatives G<br>Media communication (print)............................ H<br>Media communication (electronic) ....I<br>Street drama..................... J<br>Personal Experience....... K<br>Others..............................L<br>Not applicable.................M		

Section 4: Practice on Cholera and its vaccine

**Section 4a: Practice on cholera and its treatment/management**				
Q. No	Questions	Response Categories	Code	Direction
401	Were you or anyone of your household affected from cholera within last 6 months?	Yes...1<br>No..2<br>Don't know...9	 →<br>→	 412<br>412
401a	If yes, what was that, diarrhea or cholera?	Diarrhea.. A<br>Cholera.. B		
402	If yes, how many members of your household were affected?	______________(Nos.)		

- Please, tell me about the treatment or management of the person who affected by cholera and were present at the study area (give some idea about study area if need)
- If no member of the household was affected by cholera but a member was affected by diarrhea, then tell me about the treatment or management of the affected person
- If two or more members of a household were affected, then tell me about the treatment or management of the severe affected or most recently affected person

Q. No	Questions	Response Categories	Code	Direction
403	The name of affected person			
403 a	The PID of affected person			
404	What were the sign and symptoms?<br><br>(Multiple answers acceptable)	Passage of 3 or more loose or water stool in 24 hours ……………………..............A<br>Repeated vomiting ....B<br>Severe dehydration within very short time ....C<br>Muscles cramp ……...............................D<br>Scanty urination ....E<br>Other..F		
404a	How did you confirm that it was cholera?	Through lab diagnosis ………………...1<br>Formal health care provider's advice ....2<br>Non formal healthcare provider's advice ....…………………………....3<br>Perception of households ……………..4<br>Other………………………………....7		
405	What type of treatment was given to the patient?<br>(Multiple answers acceptable)	ORS………………………………… ..A<br>Rice saline……………………………..B<br>IV saline ………………………………C<br>Homemade saline..................................D<br>Zink tablet or syrup …………………...E<br>Antibiotics……………………………..F<br>Herbs…………………………………G<br>Homeopath……………………………H<br>Plain water……………………………..I<br>Nothing ………………………………..J<br>Others …………………………………K<br>Do not know …………………………..L		
406	Was the patient referred from home for treatment?	Yes..1<br>No...2	→	412
407	If yes, where did you seek treatment?<br>(Multiple answers acceptable)	Mohakhali cholera Hospital, icddr,b ….A<br>Mirpur cholera Hospital, icddr,b ………B<br>Medical College Hospital (public) ....C<br>Other Government hospital....................D<br>Private clinic/hospital.............................E<br>NGO clinic/hospital...............................F<br>Private chamber ....G<br>Allopathic pharmacy.............................H<br>Homeopathic pharmacy..........................I<br>Spiritual treatment place ....J<br>Herbal treatment place ....K<br>Other...L		
408	Who referred the patient?<br>(Multiple answers acceptable)	M.B.B.S Doctor .... A<br>Nurse .... B<br>Family Welfare Visitor......................... C<br>SACMO/Medical Assistant.................. D<br>Heath Assistant.....................................E<br>Family Welfare Assistant......................F<br>Paramedics .... G<br>ICVB Staff/Volunteer .... H<br>Community Health Worker (NGO)........I<br>Pharmacist/Drug sellers/ Village Doctor .... J<br>Kabiraj/Hekim...................................... K<br>Spiritual healer (Fakir) ....L<br>Homeopath Doctor .... M<br>No one .... N<br>Others (specify) .... O		

Q. No	Questions	Response Categories	Code	Direction
409	Who made decision from your household to take the patient to hospital?	Own ....1<br>Husband...2<br>Wife...3<br>Mother/ father..4<br>Mother-in-law/ Father-in-law................5<br>Other members of the family ....6<br>Other relatives ....7<br>Other (Specify)....................................77		
410	How much money was required for treatment of the patient?	a. Medicine___________ (tk)<br>b. Transportation_______ (tk)<br>c. Other______________ (tk)<br>d. Total______________ (tk)		
411	Was there any problem to reach the health facility?<br>(Multiple answers acceptable)	Transportation problem......... ....A<br>Lack of money.................................. B<br>Did not have address of<br>health facility where cholera treatment<br>is available.......................................C<br>Jam ....D<br>Other .... E<br>No problem.......................................F		
412	What measures have you undertaken to prevent cholera in your house?<br>(Multiple answers acceptable)	Use of safe water for drinking &<br>household works .... A<br>Provision of safe drinking water supply.B<br>Use of sanitary latrine/ Proper sanitary<br>disposal of excreta, refuse .... C<br>Satisfactory sewage system.................D<br>Washing hands with soap before meal.. E<br>Washing hands with after defecation . ..F<br>Protection of foods (taking hot food and<br>avoiding rotten food)...........................G<br>Housefly control measures..................H<br>Early case finding in the community for<br>the initiation of prompt treatment.........<br>..I<br>Disinfection of all the contaminated<br>articles by phenol, boiling or lime........ J<br>Good hygiene practice.........................K<br>Health education (advises to drink tube<br>well/ boiled/ tablet treated water etc.)…L<br>Taking cholera vaccine....................... M<br>Others ....N<br>Do not know ....O<br>No measures have taken ....P		

Q. No	Questions	Response Categories	Code	Direction
413	Please, mention your last experience on the following matters			
	a. Safe water was used for drinking purpose	Yes…..1<br>No …..2		
	b. Safe water was used for household works	Yes…..1<br>No …..2		
	c. Washed hand before taking last meal	Yes.. ...1<br>No 2		
	d. Used sanitary latrine	Yes... 1<br>No 2		
	e. Washed hands with soap after defecation	Yes... 1<br>No 2		
	f. Took fresh food	Yes... 1<br>No 2		
**Section 4b: Practice on cholera vaccine (if 321=2, skip this section and go to section 5)**				
414	Have you ever received Cholera vaccine?	Yes... 1<br>No 2	→<br>→	416<br>415
415	If no- why?<br>(Multiple answers acceptable)	Afraid of side effect.............................A<br>Busy with work ....B<br>Did not know about cholera vaccination schedule.. C<br>The vaccination center was far away from my home ....D<br>Not informed ....E<br>It is costly .... F<br>Do not know about address of vaccination centre ....G<br>No need of vaccine ....H<br>Others………………………………..I	→	501
416	If yes- why?	To prevent cholera...............................A<br>To keep health well ....B<br>By following others.............................C<br>Others ....D		
416a	Which cholera vaccine did you receive?	ShanChol..1<br>Dukoral...2<br>Other...7		
417	From where did you receive the vaccine?	Mohakhali cholera Hospital, icddr,b. ….1<br>Mirpur cholera Hospital, icddr,b ………2<br>Medical College Hospital (public) ....3<br>Other Government hospital.....................4<br>Private clinic/hospital.............................5<br>NGO clinic/hospital................................6<br>Private chamber ....7<br>Allopathic pharmacy.............................8<br>Homeopathic pharmacy..........................9<br>EPI centre…………..........................10<br>Not available...11<br>Other..12		
418	How many doses did you receive?	__________(Nos.)<br>Do not know 99		

Q. No	Questions	Response Categories	Code	Direction
419	If two doses, what was the interval between two doses?	__________(Days.)<br>Do not know ....99<br>Not applicable..................................88		
420	Did you like the test of the vaccine?	Yes... 1<br>No 2	→	422
421	If no- why didn't you like?<br>(Multiple answers acceptable)	a. ________________________________<br>b. ________________________________<br>c. ________________________________		
422	Did you face any health problems after vaccination?	Yes... 1<br>No 2	→	431
423	If yes- what were the problems?<br>(Multiple answers acceptable)	Headache ....A<br>Itching..B<br>Weakness...C<br>Joint pain ....D<br>Vomiting...E<br>Diarrhoea .... F<br>Others ....G		
424	How long did you suffer for this problem?	___________hours__________min		Write 00 if <1 hour
425	Did you seek treatment for the problems?	Yes... 1<br>No 2	→	431
426	If yes, what type of treatment did you take?<br>(Multiple answers acceptable)	Herbs……………………………………A<br>Spiritual…………………………………B<br>Allopathic………………………………C<br>Homeopath……………………………...D<br>Other (specify) …………………………E		
427	Who provided the treatment?<br>(Multiple answers acceptable)	M.B.B.S Doctor ....………..…..A<br>Nurse…………………………………... B<br>Family Welfare Visitor …......................C<br>SACMO/Medical Assistant……………..D<br>Heath Assistant..……….…………..……E<br>Family Welfare Assistant.………...……F<br>Paramedics……………………...……G<br>ICVB Staff/Volunteer …………………...H<br>Community Health Worker (NGO). …..I<br>Pharmacist/Drug sellers/Village Doctor<br>…………………………..……………J<br>Kabiraj/Hekimi…………………...……K<br>Fakir……………………………………L<br>Homeopath Doctor…………................M<br>Relatives…………………………..……N<br>Family Members...................................O<br>Self .... P<br>Neighbor...Q<br>Others (specify) ....R		

Q. No	Questions	Response Categories	Code	Direction
428	Did you visit any health facility for the treatment of the problem?	Yes... 1<br>No 2	→	431
429	If yes, where did you visit?<br>(Multiple answers acceptable)	Mohakhali cholera Hospital, icddr,…….A<br>Mirpur cholera Hospital, icddr,b ………B<br>Medical College Hospital (public) ....C<br>Other Government hospital......................D<br>Private clinic/hospital...............................E<br>NGO clinic/hospital..................................F<br>Private chamber ....G<br>Allopathic pharmacy................................H<br>Homeopathic pharmacy............................I<br>Spiritual treatment place ....J<br>Other...K		
430	How much money was required for treatment of the patient?	a. Medicine___________ (tk)<br>b. Transportation_______ (tk)<br>c. Other______________ (tk)<br>d. Total______________ (tk)		
431	Who encouraged you to take this vaccine?	M.B.B.S Doctor ....………..01<br>Nurse…………………………………… 02<br>Family Welfare Visitor……..................03<br>SACMO/Medical Assistant……….…..04<br>Heath Assistant………………….....……05<br>Family Welfare Assistant……………...06<br>Paramedics…………………….……07<br>ICVB Staff/Volunteer ………………...08<br>Community Health Worker (NGO)…...09<br>Pharmacist/Drug sellers/Village Doctor<br>……………………….……………10<br>Kabiraj/Hekim…………………..……..11<br>Fakir……………………………………12<br>Homeopath Doctor…………….............13<br>Relatives………………………….……14<br>Family Members………………………15<br>Self……………………………….……16<br>Neighbour…………………..…………17<br>Others (specify)………………………..77		

Section 5: Attitude towards Cholera and cholera vaccine

Please, give your opinion how much you agree on the following statements				
**Q. No**	**Questions**	**Response Categories**	**Code**	**Direction**
501	It may cause disease if stool is passed at anywhere	Fully agreed... 1<br>Partially agreed.................................... 2<br>Disagreed... 3		
502	We should wash our hands before having food	Fully agreed... 1<br>Partially agreed.................................... 2<br>Disagreed... 3		
503	We should wash our both hands by soap or ash after defecation	Fully agreed... 1<br>Partially agreed.................................... 2<br>Disagreed... 3		

Q. No	Questions	Response Categories	Code	Direction
504	Cholera is a severe health problem which may cause death	Fully agreed.. 1<br>Partially agreed.................................... 2<br>Disagreed.. 3		
505	Cholera is very serious for adults	Fully agreed.. 1<br>Partially agreed.................................... 2<br>Disagreed.. 3		
506	Cholera is very serious for children	Fully agreed.. 1<br>Partially agreed.................................... 2<br>Disagreed.. 3		
507	Cholera is common in the community	Fully agreed.. 1<br>Partially agreed.................................... 2<br>Disagreed.. 3		
508	Cholera can be prevented through vaccination	Fully agreed.. 1<br>Partially agreed.................................... 2<br>Disagreed.. 3		
509	Cholera vaccine may be harmful for health	Fully agreed.. 1<br>Partially agreed.................................... 2<br>Disagreed.. 3	→	511
510	If agreed, why do you think so?	a.____________________<br>b.____________________<br>c.____________________		
511	We should encourage people for cholera vaccination	Fully agreed.. 1<br>Partially agreed.................................... 2<br>Disagreed.. 3		

Section 6: Recommendation for ICVB project

Q. No	Questions	Response Categories	Code	Direction
601	Do you have any suggestion for a successful implementation of ICVB project?	Yes.. 1<br>No 2	→	Section 7
602	If yes, what are those suggestions?	a. ____________________________________<br>b. ____________________________________<br>c. ____________________________________		

Section 7: Other observations/Note (If you do not have anything to note down, please skip to q801)

Final Section

Q. No	Questions	Response Categories	Code	Direction
801	Date of data collection	......./ ..../ 201____<br>day/ month/year		
802	Start time of interview	.... / ....<br>Hour / minute		Railway time
803	End time of interview	.... / ....<br>Hour / minute		Railway time
